# Structural and biochemical characterization of the pleckstrin homology domain of the RhoGEF P-Rex2 and its regulation by PIP_3_

**DOI:** 10.1016/j.yjsbx.2018.100001

**Published:** 2018-12-10

**Authors:** Jennifer N. Cash, Prateek V. Sharma, John J.G. Tesmer

**Affiliations:** a Department of Pharmacology, Life Sciences Institute, University of Michigan, Ann Arbor, MI 48109-2216, USA; b Department of Biological Chemistry, Life Sciences Institute, University of Michigan, Ann Arbor, MI 48109-2216, USA; c Department of Biological Sciences and of Medicinal Chemistry and Molecular Pharmacology, Purdue University, West Lafayette, IN 47904, USA

**Keywords:** DEP, dishevelled, Egl-10, and pleckstrin, DH, Dbl homology, DSF, differential scanning fluorimetry, DTT, dithiothreitol, EDTA, ethylenediaminetetraacetic, Gβγ, G protein β and γ subunits, Ins(1,3,4,5)*P*_4_, inositol-1,3,4,5-tetrakisphosphate, IP4P, inositol polyphosphate 4-phosphatase, MBP, maltose binding protein, PDZ, post-synaptic density protein, *Drosophila* disc large tumor suppressor, and zonula occludens-1 protein, PH, pleckstrin homology, PIP_3_, phosphatidylinositol 3,4,5-trisphosphate, PMSF, phenylmethylsulfonyl fluoride, P-Rex, phosphatidylinositol 3,4,5-trisphosphate-dependent Rac exchanger, PTEN, phosphatase and tensin homolog, RhoGEF, Rho guanine-nucleotide exchange factor, Pleckstrin homology domain, Rho guanine nucleotide exchange factor, Phosphatidylinositol 3,4,5-trisphosphate, P-Rex

## Abstract

P-Rex family Rho guanine-nucleotide exchange factors are important regulators of cell motility through their activation of a subset of small GTPases. Both P-Rex1 and P-Rex2 have also been implicated in the progression of certain cancers, including breast cancer and melanoma. Although these molecules display a high level of homology, differences exist in tissue distribution, physiological function, and regulation at the molecular level. Here, we sought to compare the P-Rex2 pleckstrin homology (PH) domain structure and ability to interact with PIP_3_ with those of P-Rex1. The 1.9 Å crystal structure of the P-Rex2 PH domain reveals conformational differences in the loop regions, yet biochemical studies indicate that the interaction of the P-Rex2 PH domain with PIP_3_ is very similar to that of P-Rex1. Binding of the PH domain to PIP_3_ is critical for P-Rex2 activity but not membrane localization, as previously demonstrated for P-Rex1. These studies serve as a starting point in the identification of P-Rex structural features that are divergent between isoforms and could be exploited for the design of P-Rex selective compounds.

## Highlights

• The crystal structure of the P-Rex2 PH domain was determined to 1.9 Å.
• In comparison to the P-Rex1 PH, the β5/β6 loop conformation is the most divergent.
• P-Rex2 and P-Rex1 PH domains interact with Ins(1,3,4,5)*P*_4_ similarly.
• PIP_3_ binding is required for P-Rex2 activation but not membrane localization.

## Graphical abstract

**Figure:**
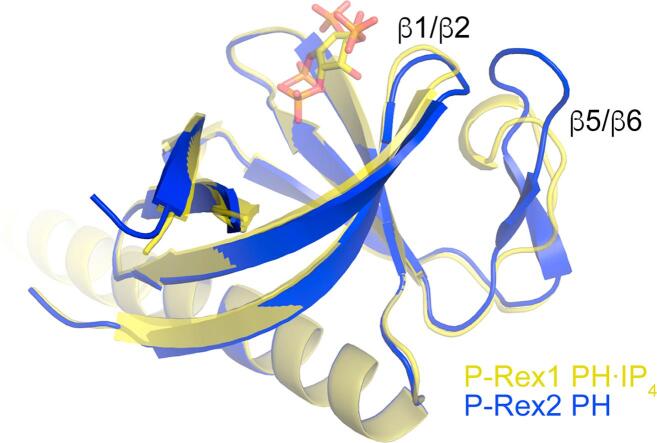

## 1 Introduction

Rho guanine-nucleotide exchange factors (RhoGEFs) of the Dbl family, comprised of around 70 members, are critical regulators of signaling by small GTPases such as Rac, Cdc42, and Rho (Aittaleb et al., 2010, Cook et al., 2014). Within this family, the phosphatidylinositol 3,4,5-trisphosphate (PIP_3_)-dependent Rac exchanger (P-Rex) subfamily RhoGEFs P-Rex1 and P-Rex2 act as important regulators of cell migration (Welch, 2015). P-Rex1 is mainly expressed in neutrophils and the brain and is responsible for functions such as neutrophil migration, production of reactive oxygen species, and neurite differentiation (Waters et al., 2008, Welch et al., 2002, Welch et al., 2005). P-Rex2 is more widely expressed, and it has been shown to play an important role in Purkinje cell morphogenesis and motor coordination (Donald et al., 2004, Donald et al., 2008). P-Rex1 and P-Rex2 have been knocked out in mouse models, both separately and in combination, and this results in overall healthy mice with mild symptoms that result from neutrophil defects and/or deficiencies in motor coordination (Donald et al., 2008, Dong et al., 2005, Welch et al., 2005). Interestingly, both isoforms have been implicated in human cancers, wherein they act as pro-metastatic factors. P-Rex1 has been shown to be overexpressed in prostate cancer, breast cancer, and melanoma, and this is associated with tumor metastasis and poor patient outcome (Lindsay et al., 2011, Montero et al., 2011, Qin et al., 2009, Sosa et al., 2010). P-Rex2 is commonly mutated in breast cancer and melanoma, with mutations distributed throughout the length of the protein (Berger et al., 2012, Nik-Zainal et al., 2016). One study identified *PREX2* as one of the most mutated genes in human metastatic melanomas (Berger et al., 2012). Of these mutations tested, most appear to be activating, resulting in increased tumor incidence and decreased survival rates (Berger et al., 2012, Deribe et al., 2016). Understanding how these proteins are regulated at the molecular level is thus an important step toward identifying their function in cancer and how one might target them therapeutically.

**Fig. 1:**
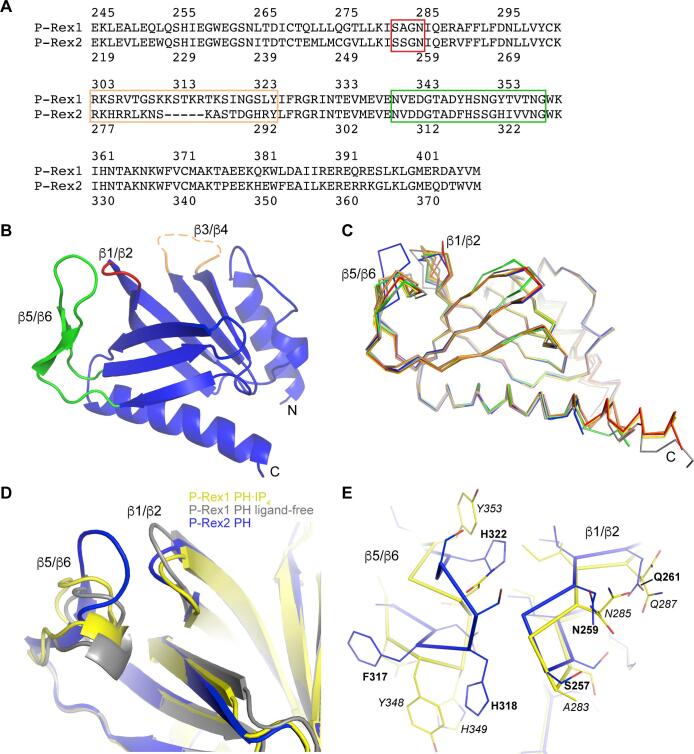
Structural comparison of the P-Rex2 and P-Rex1 PH domains. (A) Protein sequence alignment of the P-Rex1 and P-Rex2 PH domains with the labeled loops in (B) shown boxed in corresponding colors. (B) Ribbon diagram representation of the structure of the P-Rex2 PH domain (PDB: 6BNM). N- and C-termini are labeled, as well as loops that are discussed in the main text. The anti-parallel β-strands of the core fold are sequentially numbered, and the loops decorating this core are named by the strands they connect. The dashed line indicates an unstructured loop. (C) Structural alignment of all of the available P-Rex1 PH domain structures (PDB: 5D27, PDB: 5D3V, PDB: 5D3W, PDB: 5D3X, and PDB: 5D3Y) with the P-Rex2 PH domain structure (blue), which highlights conformational differences in the β1/β2 and β5/β6 loops. Domains were aligned in PyMOL. (D) The P-Rex1 PH domain from an Ins(1,3,4,5)*P*_4_ -bound structure (5D3X chain A, yellow) and the P-Rex1 PH domain structure with nothing bound in the PIP_3_ site (5D27, grey) are shown aligned with the P-Rex2 PH structure (blue). (E) Alignment shown in (D), but without the ligand-free P-Rex1 PH domain, zoomed in on the β1/β2 and β5/β6 loop region. Select homologous residues between P-Rex1 (italics) and P-Rex2 (bold) are labeled to serve as reference points. There are substantial mainchain Cα position deviations for some of the residues here. For example, P-Rex2 His318 and Ser319 are 4.9 Å and 8.3 Å away from P-Rex1 His349 and Ser350, respectively. (For interpretation of the references to colour in this figure legend, the reader is referred to the web version of this article.)

At their N-termini, P-Rex proteins contain Dbl homology (DH)/pleckstrin homology (PH) domains, a tandem arrangement found in nearly all Dbl family RhoGEFs. These domains are referred to as the catalytic core, although the DH domain harbors the nucleotide exchange activity of the enzyme. Following the DH/PH module are two dishevelled, Egl-10, and pleckstrin (DEP) domains and then two post-synaptic density protein, *Drosophila* disc large tumor suppressor, and zonula occludens-1 protein (PDZ) domains, ending with a large, C-terminal inositol polyphosphate 4-phosphatase (IP4P)-like domain with no identified enzymatic activity (Welch et al., 2002). Evidence in the literature suggests that the accessory domains C-terminal to the DH domain contribute to autoinhibition, as removing them results in increased RhoGEF activity (Hill et al., 2005). However, lack of data on the ternary structure of the domains outside of the catalytic core (Cash et al., 2016, Lucato et al., 2015) and the molecular details of how they interact with the DH/PH tandem limits our understanding of this autoregulation. P-Rex1 and P-Rex2 are overall 58% identical (alignment of the PH domains is shown in Fig. 1A), with most sequence divergence occurring in the IP4P domain. Both enzymes are synergistically activated by PIP_3_ and heterotrimeric G protein βγ heterodimers (Gβγ) (Barber et al., 2007, Hill et al., 2005, Li et al., 2005, Mayeenuddin et al., 2006, Urano et al., 2008, Welch et al., 2002). Previously, we characterized the molecular interaction of the P-Rex1 PH domain with inositol-1,3,4,5-tetrakisphosphate [Ins(1,3,4,5)*P*_4_], a soluble analog of PIP_3_ (Cash et al., 2016). We determined that PIP_3_ binding to the PH domain is absolutely required for P-Rex1 activity in cells but is dispensable for its membrane localization, leading to the hypothesis that PIP_3_ allosterically activates P-Rex1 by inducing a conformational change upon binding. In contrast, the exact location of the Gβγ binding site and its mechanism of activation have not been elucidated.

P-Rex proteins have been shown to be regulated by other molecules in addition to PIP_3_ and Gβγ. For example, the regulatory subunit of type I PKA interacts with P-Rex1 through the PDZ domains, whereby P-Rex1 localizes PKA to the plasma membrane, and PKA stimulation causes phosphorylation and inactivation of P-Rex1 (Chávez-Vargas et al., 2016). P-Rex1 has also been shown to be activated and localized to the cell membrane by Norbin, a G protein-coupled receptor adaptor protein, through the PH domain (Pan et al., 2016). In some cases, P-Rex regulatory interactions are restricted to a particular isoform. P-Rex2 has been shown to interact with the tumor suppressor phosphatase and tensin homolog (PTEN) in a co-inhibitory fashion, a relationship that has been shown to be specific to P-Rex2 over P-Rex1 (Hodakoski et al., 2014, Mense et al., 2015). We have shown that P-Rex2 associates with HEK293T cell membranes at higher levels than P-Rex1, albeit through an unknown mechanism (Cash et al., 2016). We went on to show that residue divergence in the β3/β4 loop of the PH domain results in a more positively charged loop in P-Rex1 that acts as a nonspecific membrane localization element. When this loop sequence is swapped into P-Rex2, it leads to even greater membrane association (Cash et al., 2016).

To investigate whether the P-Rex2 PH domain has structural differences from that of P-Rex1 that may underlie some of the aforementioned functional disparities between P-Rex1 and P-Rex2, we determined the crystal structure of the P-Rex2 PH domain to 1.9 Å resolution. This structure revealed the most prominent difference to be in the conformation of the β5/β6 loop, a region proposed to be a regulatory protein-protein interaction site in P-Rex1 (Cash et al., 2016). Biochemical and cell-based assays suggest that although the residues involved in PIP_3_ binding are the same in P-Rex1 and P-Rex2, PIP_3_ binding may be relatively less important to P-Rex2 activation.

## 2 Material and methods

### 2.1 Cloning

Human P-Rex1 cDNA was a gift from Dr. James Garrison (University of Virginia). Human P-Rex2 cDNA was purchased from Addgene (plasmid #41555). DNA encoding the P-Rex1 PH domain (residues 245–408) or P-Rex2 PH domain (residues 219–377 for crystallography, 219–364 for assays) was cloned into a modified pMAL expression vector (pMALc2H_10_T) (Kristelly et al., 2004). P-Rex2 (residues 1–1606) was fused with EGFP in the pEGFP-C1 vector (Clontech Laboratories, Inc.). Site directed mutations were created using QuikChange (Qiagen) and confirmed by DNA sequencing.

### 2.2 Protein production and purification

Rosetta (DE3) pLysS *E. coli* cells (Novagen) were used to overexpress P-Rex PH constructs as N-terminally 10xHis-tagged maltose binding protein (MBP)-fusion proteins. Cells were grown in Terrific Broth plus carbenicillin to an OD_600_ of 0.8, and then protein expression was induced with 0.1 mM isopropylthiogalactopyranoside at 20 °C for 18 h. After harvesting, cells were lysed in a buffer containing 20 mM HEPES pH 8, 100 mM NaCl, 2 mM dithiothreitol (DTT), and protease inhibitors [0.1 mM ethylenediaminetetraacetic acid (EDTA), 0.001 mM leupeptin, 1 mM lima bean trypsin inhibitor, and 0.1 mM phenylmethylsulfonyl fluoride (PMSF)], and then recombinant protein was extracted using histidine affinity (Ni-NTA) resin chromatography. Proteins were eluted from the Ni-NTA resin in a buffer containing 20 mM HEPES pH 8, 200 mM NaCl, 2 mM DTT, and 250 mM imidazole. MBP-PH proteins were then simultaneously dialyzed into buffer containing 20 mM HEPES pH 8, 100 mM NaCl, and 2 mM DTT and treated with a 1:1 M ratio of TEV protease to remove the N-terminal MBP tag, which was then removed by re-passage over Ni-NTA resin. Final purity was achieved by processing the PH domains over an S75 size exclusion column (GE Healthcare) in a buffer containing 20 mM HEPES pH 8, 200 mM NaCl, and 2 mM DTT.

### 2.3 Crystallization, data collection, and structure determination

Initial P-Rex2 PH domain crystallization conditions were determined using the Index HT screen (Hampton Research) in a sitting drop format. Optimized crystals were produced by the hanging-drop method from drops containing 0.5 μl protein plus 0.5 μl well solution suspended over 1 ml well solution at 20 °C. The P-Rex2 PH domain was crystallized using 6.2 mg/ml PH domain and a well solution containing 100 mM sodium acetate trihydrate pH 5 and 3 M NaCl. Crystals were transferred into a solution containing 10 mM HEPES pH 8, 100 mM NaCl, and 1 mM DTT plus well solution supplemented with 15% glycerol and then frozen in liquid N_2_. Diffraction data were collected at 110 K on a CCD detector at beamline 21-ID-D at the Advanced Photon Source. Data were integrated and scaled using HKL2000 (Otwinowski and Minor, 1997), and initial phases were provided by molecular replacement using PHASER (Mccoy et al., 2007, Winn et al., 2011) with the P-Rex1 PH (PDB: 5D27) as a search model. The atomic model was built using manual building in Coot (Emsley et al., 2010) alternating with maximum-likelihood refinement in Refmac5 (Murshudov et al., 1997), and the structure was validated using MolProbity (Chen et al., 2010). Data collection and refinement statistics are shown in Table 1. Coordinates and the supporting experimental data have been deposited in the Protein Data Bank with the accession number 6BNM. Structure images were rendered using The PyMOL Molecular Graphics System, Version 1.8.0.3 Schrödinger, LLC. SSM Superposition in Coot was used to calculate root-mean-square-deviation values (Krissinel and Henrick, 2004).

**Table 1:** Crystallographic data collection and refinement.

Data Collection	P-Rex2 PH
Wavelength (Å)	1.078
Resolution range (Å)	50–1.90 (1.93–1.90) a
Space group	*P*3_1_21
Cell dimensions	
*a, b, c* (Å)	60.1, 60.1, 86.2
α, β, γ (°)	90, 90, 120
Total reflections	105,136
Unique reflections	14,598
Multiplicity	7.2 (4.5)
Completeness (%)	99.0 (96.8)
*I/σI*	28.4 (1.5)
*R_merge_* (%)	6.4 (71.3)
CC_1/2_	72.6

*Refinement*	
Number of molecules per asymmetric unit	
Protein	1
Ligand/ion	4 Cl^−^
Number of atoms	
Protein	1135
Ligand/ion	4
Water	74
*R_work_/R_free_* (%)	19.3/23.2 (28.6/30.7)
Rmsd	
Bond lengths (Å)	0.011
Bond angles (°)	1.45
Average B-factor (Å^2^)	
Protein	36.1
Ligand/ion	34.6
Water	40.6
Ramachandran Analysis^b^	
Favored (%)	97.7
Outliers (%)	0
PDB ID	6BNM

a Values in parentheses represent the highest-resolution shell.

b As defined in MolProbity.

### 2.4 Differential scanning fluorimetry (DSF)

Thermal denaturation assays were performed using a QuantStudio 7 Flex instrument (Applied Biosystems). Ins(1,3,4,5)*P*_4_ (D-*myo*-Inositol-1,3,4,5-tetraphosphate) was purchased from Cayman Chemical. Proteins were diluted to 0.1 mg/ml into a buffer containing 50 mM HEPES pH 8, 150 mM NaCl, 2 mM DTT, and SYPRO orange (Thermo Fisher Scientific) was used at a final concentration of 4× in a volume of 10 μl in a 384-well PCR plate (Applied Biosystems). Experiments were performed three times in duplicate. Melting temperatures were determined by monitoring the fluorescence change of SYPRO orange as it binds to unfolded protein in the presence or absence of 1 mM Ins(1,3,4,5)*P*_4_. Melting curves were analyzed by fitting to a Boltzmann model using Protein Thermal Shift Software v1.3 (Applied Biosystems).

### 2.5 Isothermal titration calorimetry (ITC)

ITC experiments were performed in a Nano-ITC Low volume calorimeter (TA Instruments). Five hundred microliters of P-Rex2 PH domain (70 μM) in a buffer of 20 mM HEPES pH 8 plus 150 mM NaCl was added to the cell and 50 μl of Ins(1,3,4,5)*P*_4_ (300 μM) in the same buffer was taken in the syringe. The experiments were performed at 25 °C with 2 μl Ins(1,3,4,5)*P*_4_ injected into the P-Rex2 PH domain every 300 s a total of 25 times with a stirring speed of 250 rpm. The *K_D_* and ΔH of the reactions were calculated using the Launch NanoAnalyze software (TA Instruments). Experiments were performed three times, and the average is shown along with the standard deviation of the mean.

### 2.6 Fluorescence polarization (FP)

FP experiments were performed three times in duplicate, with a reaction volume of 25 μl per well in a 384-well, black, low-volume plate with round-bottom wells. Proteins were diluted to 200 nM into a buffer containing 50 mM HEPES pH 8, 150 mM NaCl, 2 mM DTT. Ins(1,3,4,5)*P*_4_ was added in varying concentrations to build a dose response curve, followed by 15 nM BODIPY TMR-PIP_3_ (#C-39M6, Echelon Biosciences). This mixture was incubated for 45 min at room temperature prior to reading fluorescence polarization at 542/574 nm on a FlexStation 3 microplate reader (Molecular Devices). Data were analyzed using nonlinear regression in GraphPad Prism 7 and fit using a competition binding equation with one site homologous binding without consideration of ligand depletion.

### 2.7 Cell culture and transfection

HEK293T cells were maintained in high glucose Dulbecco’s Modified Eagle’s Medium supplemented with 10% fetal bovine serum plus 100 U/ml penicillin and 100 μg/ml streptomycin and incubated at 37 °C in a humidified atmosphere with 6% CO_2_. Cells were transfected with DNA using FuGENE 6 (Promega) in Opti-MEM according to the manufacturer’s instructions.

### 2.8 Membrane localization assay

HEK293T cells were plated at 0.2 × 10^6^ cells/well in 12-well cell culture plates. Each well was transfected with 1 μg EGFP-tagged P-Rex2 construct DNA. EGFP, which is cytosolic, and EGFP-LARG-2xPH, which strongly localizes to the cell membrane (Aittaleb et al., 2009), served as negative and positive controls, respectively. Cells were harvested approximately 24 h later by scraping into PBS, centrifuging at 3000 × g, and then resuspending the pellet in 100 μl lysis buffer (20 mM HEPES pH 8, 20% glycerol, 2 mM DTT, 0.1 mM EDTA, 0.001 mM leupeptin, and 1 mM lima bean trypsin inhibitor). Cells were lysed by carrying out three cycles of flash freezing in liquid nitrogen, thawing on ice, and vortexing. To begin to separate membrane-associated protein from cytosolic, lysates were thawed and 30 μl lysis buffer added before treating with 0.2 μl benzonase (Sigma E1014) for 10 min at 20 °C. The lysates were then pre-cleared by centrifugation at 400× g for 10 min at 4 °C. Supernatant was removed, reserving 10 μl of a total lysate sample, and then ultracentrifuged 45 min at 50,000 RPM to pellet the membrane fraction. The resulting supernatant was removed and the pellet resuspended in 10 μl lysis buffer supplemented to 300 mM NaCl. Total lysate, supernatant, and membrane pellet samples (10 μl each) were loaded into low-volume black 384-well plates and the fluorescence was read (488/520 nm) in a FlexStation 3 microplate reader (Molecular Devices). EGFP-tagged protein associated with either the membrane or cytosolic fraction was represented as a percent of the total EGFP-tagged protein in the total lysate sample.

### 2.9 Luciferase-reporter gene assay

pSRE.L firefly and pRL-thymidine kinase (pRL-TK) Renilla luciferase-reporter gene constructs were described previously (Evelyn et al., 2007). Briefly, luciferase activity from the SRE.L reporter gene corresponds to serum response factor activation by RhoA, Rac, and Cdc42, thus acting as a readout of activation of these GTPases. HEK293T cells were seeded at 0.03 × 10^6^ cells/well in 96-well cell culture plates. Each well was transfected with 25 ng pSRE.L and 20 ng pRL-TK along with EGFP-tagged P-Rex2 construct or EGFP-LARG-2xPH DNA, and total DNA was normalized using empty pRK5 vector. Plates were assayed approximately 26 h after transfection using the Dual-Glo Luciferase Assay System (Promega) according to manufacturer’s protocol and a SpectraMax M5 plate reader (Molecular Devices). Samples were transferred from the cell culture plates to grey 96-well plates (PerkinElmer) before readings. In these experiments, there was no significant activation of the pSRE.L reporter gene in the absence of transfected P-Rex2 DNA, indicating little to no endogenous P-Rex2.

### 2.10 In-gel imaging of EGFP-tagged P-Rex2 constructs

To assess expression levels of P-Rex2 constructs, in-gel imaging was performed on EGFP-tagged constructs from the cells harvested in the membrane localization experiments. Cells were lysed as indicated previously, and 12 μl of each total lysate sample was run on SDS-PAGE (10% polyacrylamide, samples were not boiled). The gel was washed with water three times for 5 min each and imaged with a Typhoon™ 9410 Variable Mode Imager (GE Healthcare) where fluorescence was read at 488/526 nm and 100 μm resolution. Precision Plus Protein Kaleidoscope Standard (BioRad #1610375) was used as a marker.

### 2.11 Statistical analysis

Data were analyzed using GraphPad Prism 7. Error bars indicate 95% confidence intervals. Significance was examined using a student’s *t*-test two-tailed *P*-value.

## 3 Results

### 3.1 Crystal structure of the P-Rex2 PH domain

To clarify the molecular basis of regulatory differences between P-Rex1 and P-Rex2, we decided to focus on the PH domain, which is the regulatory binding site for PIP_3_ (Cash et al., 2016). We first determined the crystal structure of the P-Rex2 PH domain to 1.9 Å resolution (Fig. 1B and Table 1). The core of the domain exhibits a canonical PH domain fold composed of a 7-stranded β-sandwich structure that is open on one side and capped on the other by a C-terminal α-helix. This core is decorated by variable loops and a short N-terminal helix that would be continuous with the C-terminal helix of the preceding DH domain. The open side of the domain is the expected location of the PIP_3_-binding site, as has been previously demonstrated in P-Rex1 (PDB: 5D3X and PDB: 5D3Y) (Cash et al., 2016). In the P-Rex2 PH domain structure, the PIP_3_-binding site is occupied by a chloride ion (Sup. Fig. 1B), which is coordinated, in part, by basic residues that line the site. In the P-Rex1 PH domain structures, negatively charged molecules from the crystallization solutions were commonly observed in the PIP_3_-binding site, including citrate and sulfate (PDB: 5D3V and PDB: 5D3W, respectively).

Despite 72% sequence identity between the PH domains of P-Rex1 and P-Rex2, there are prominent structural differences between them (Fig. 1A and C). In P-Rex1, the middle section of the β3/β4 loop is longer and more positively charged (Fig. 1A and B, orange) and has been previously demonstrated to be a nonspecific membrane localization element (Cash et al., 2016). This loop is disordered in all P-Rex1 and P-Rex 2 PH domain structures determined to date. The β1/β2 loop, which contributes to the PIP_3_-binding site, has previously been observed in P-Rex1 in a variety of conformations, undoubtedly influenced by occupancy of the pocket (Fig. 1C). For example, the β1/β2 loop of a P-Rex1 PH structure with the PIP_3_-binding site empty moves away from the pocket (Fig. 1D, PDB: 5D27) (Cash et al., 2016). The conformation of the P-Rex2 β1/β2 loop is most similar to that seen in the Ins(1,3,4,5)*P*_4_-fully occupied P-Rex1 PH domain with an overall core root-mean-square-deviation of 0.72 Å for the two structures of the domains (compared to 5D3X chain A; Fig. 1D), and this conformation could be influenced by either the bound chloride or the unique structure of the β5/β6 loop (Fig. 1C and D). The P-Rex2 β5/β6 loop takes on a configuration distinct from P-Rex1 and packs in close proximity to the β1/β2 loop (Fig. 1D and E). There is a crystal contact formed at this site (Sup. Fig. 1A, cyan), but the interactions are weak (Sup. Fig. 1B). About half of these occur at the base of the β5/β6 loop, but this region is not structurally divergent from P-Rex1, so these are unlikely to confer the different conformation of the remainder of the P-Rex2 β5/β6 loop. Three additional lattice contacts could form weak hydrogen bonds. Interestingly, nitrogen NE2 of His322 (Tyr353 in P-Rex1) forms a hydrogen bond with the backbone carbonyl of Gly258, located in the β1/β2 loop. It has been proposed that P-Rex1 Tyr353 and residues on the β1 and β2 strands form a potential protein-protein interaction site that may serve important regulatory purposes in P-Rex1 (Cash et al., 2016). The structural divergence here may therefore underlie some of the unique regulatory mechanisms of P-Rex2.

### 3.2 Characterization of Ins(1,3,4,5)*P*_4_ binding to P-Rex PH domain variants

**Fig. 2:**
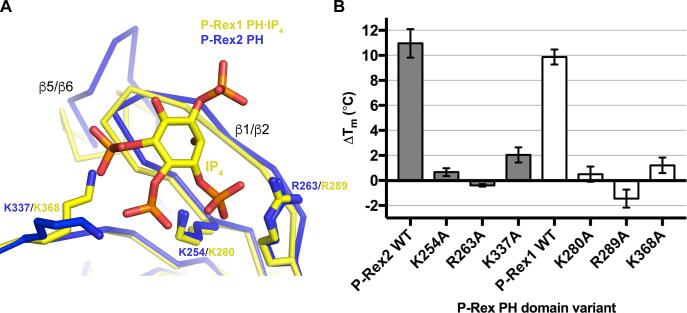
Mutations in PIP_3_-binding residues similarly reduce IP_4_ binding to the PH domains of P-Rex2 and P-Rex1. (A) Alignment of the P-Rex2 PH domain (blue) with the P-Rex1 PH domain bound to Ins(1,3,4,5)*P*_4_ (5D3X chain A, yellow) showing the side chains of basic residues that contribute to binding. (B) DSF performed with wild-type P-Rex PH domains and variants. Changes in melting temperatures (ΔT_m_) in the presence of 1 mM Ins(1,3,4,5)*P*_4_ were determined. Experiments were performed three times in duplicate, and error bars represent 95% confidence intervals. *P* < 0.0001 for all constructs compared to wild-type. (For interpretation of the references to colour in this figure legend, the reader is referred to the web version of this article.)

In order to directly compare binding of Ins(1,3,4,5)*P*_4_ to the P-Rex1 and P-Rex2 PH domains, we attempted to crystallize P-Rex2 PH·Ins(1,3,4,5)*P*_4_. However, we were not able to determine crystallization conditions for the P-Rex2 PH domain that would permit Ins(1,3,4,5)*P*_4_ binding. Instead, we examined the relative contributions of P-Rex2 K254, R263, and K337 to binding Ins(1,3,4,5)*P*_4_. These residues were chosen based on the solved P-Rex1 PH·Ins(1,3,4,5)*P*_4_ structures and sequence homology. We initially attempted to use a fluorescence polarization competition assay to analyze binding, however, PH domain variants with mutations at these positions have too low affinity for fluor-conjugated PIP_3_ to produce sufficient signal in our assay. Therefore, we used differential scanning fluorimetry to measure the effects of mutation of these residues on Ins(1,3,4,5)*P*_4_ binding. Melting temperatures of the tested variants in the absence of ligand are not significantly different from wild-type, confirming that these proteins are properly folded (Sup. Fig. 2). Wild-type P-Rex2 PH domain shows a robust increase in melting temperature of 11 °C upon binding to Ins(1,3,4,5)*P*_4_, from 44.7 °C to 55.7 °C (Fig. 2). In contrast, mutation of any one of the PIP_3_-binding residues drastically reduces the temperature shift by Ins(1,3,4,5)*P*_4_. In fact, R263A shows destabilization in the presence of 1 mM Ins(1,3,4,5)*P*_4_, possibly due to an overall increase in ionic strength in this condition. P-Rex1 variants display the same trends in binding deficiency, supporting that the PH domains of P-Rex1 and P-Rex2 bind PIP_3_ similarly.

### 3.3 Comparison of PIP_3_ affinity for P-Rex PH domains

**Fig. 3:**
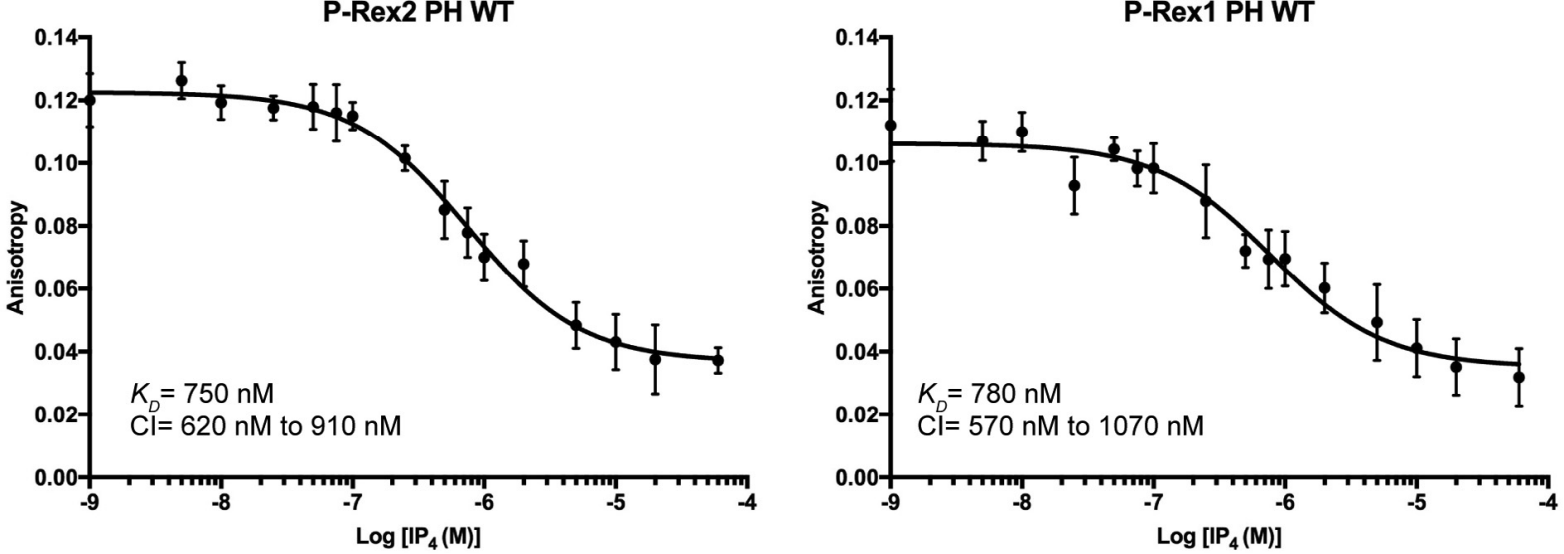
PIP_3_ analog binds P-Rex2 and P-Rex1 PH domains with similar affinity. Fluorescence polarization competition binding experiments with P-Rex PH domains and TMR-PIP_3_, titrating in Ins(1,3,4,5)*P*_4_. Experiments were performed three times in duplicate. Error bars represent 95% confidence intervals. Effective *K_D_* values are shown along with their corresponding 95% confidence interval ranges.

Using isothermal titration calorimetry (ITC), we determined the *K_D_* of Ins(1,3,4,5)*P*_4_ for P-Rex2 to be 240 ± 140 nM (Sup. Fig. 3), similar to that measured for the PH domain of P-Rex1 (440 nM) (Cash et al., 2016). We also developed a fluorescence polarization competition assay using BODIPY TMR-PIP_3_ and Ins(1,3,4,5)*P*_4_ to compare affinities. The advantages of this assay are that it provides similar information to ITC but uses relatively little protein and can be done in a 384-well, high-throughput format. Using purified, wild-type PH domains, the effective *K_D_* of BODIPY TMR-PIP_3_ was determined to be 750 nM for P-Rex2 and 780 nM for P-Rex1, again indicating no significant difference in affinity between them (Fig. 3).

### 3.4 Contribution of PIP_3_-binding residues to P-Rex2 membrane localization and activity

**Fig. 4:**
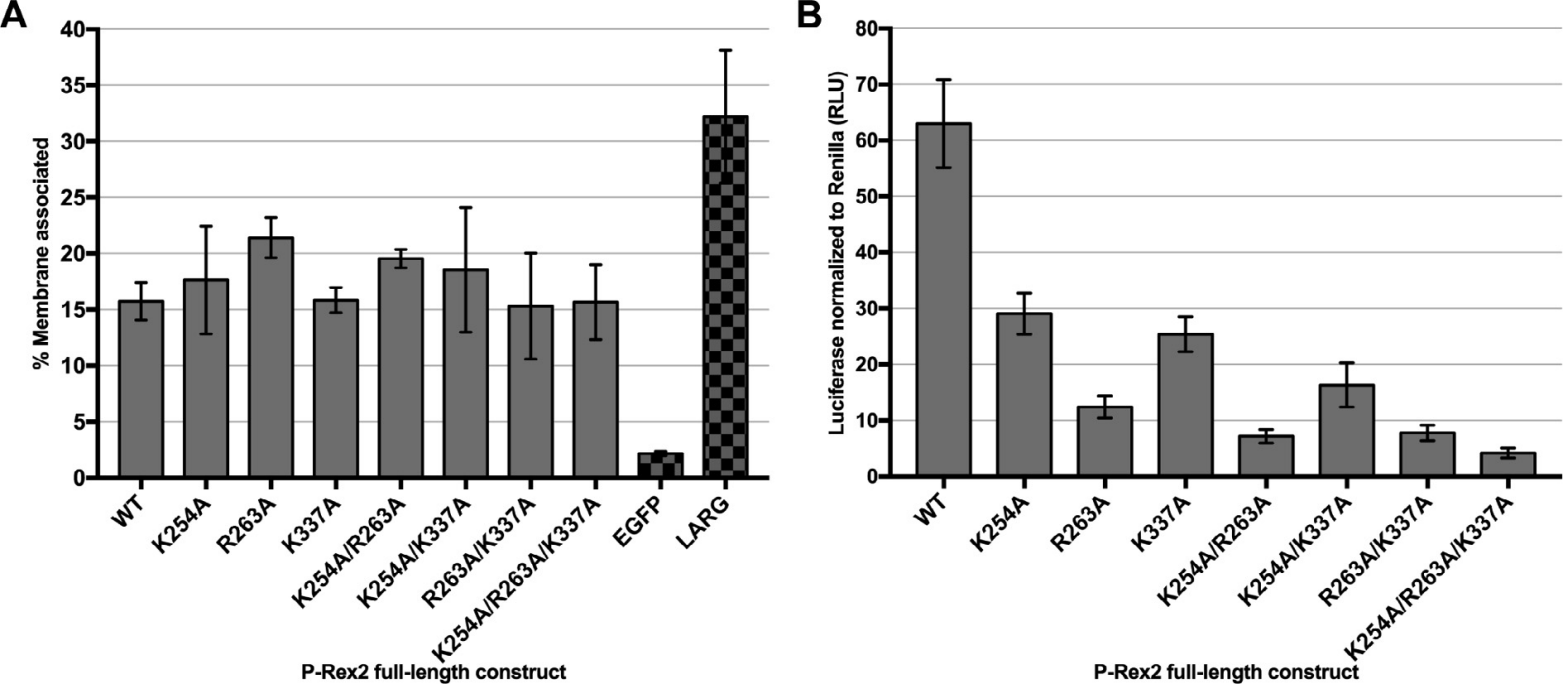
Mutations in the PIP_3_-binding site do not diminish P-Rex2 membrane localization but greatly reduce P-Rex2 activity in cells. (A) EGFP-tagged P-Rex2 variants were expressed in HEK293T cells, and membrane- and cytosol- associated proteins were separated. EGFP-tagged protein in each fraction was quantified by fluorescence and normalized to total EGFP-tagged protein expressed. Shown as controls are EGFP, which is cytosolic, and a LARG molecule engineered to strongly associate with the cell membrane. Data shown represent the average of at least three experiments performed in duplicate. Error bars indicate 95% confidence intervals. (B) Full-length P-Rex2 wild-type and variants were transiently transfected into HEK293T cells in titrations, and luciferase-reporter gene assays were performed. See also Supplementary Fig. 5. Data from the 20 ng amount of transfected P-Rex2 DNA are shown here. Experiments were performed four or more times in triplicate. Error bars represent 95% confidence intervals for the data. *P* < 0.0001 for all constructs compared to wild-type.

It has been shown previously that PIP_3_ binding is dispensable for P-Rex1 membrane localization *in vitro* but critical to its activity in cells (Cash et al., 2016). To investigate if P-Rex2 is similarly regulated by PIP_3_, we mutated residues in P-Rex2 important for PIP_3_ binding, based on homology to P-Rex1, and tested the effects on full-length enzyme in terms of its membrane localization and activity in cells. To do this, we utilized an EGFP-tagged P-Rex2 construct transiently transfected into HEK293T cells. To examine membrane localization of P-Rex2 variants, the cytosolic and membrane fractions were separated by ultracentrifugation and the amount of P-Rex2 in each was quantified based on fluorescence of the EGFP tag. To also monitor expression of the entire construct and not just EGFP alone, samples were run on SDS-PAGE and bands imaged at 488/526 nm, confirming expression of full-length, tagged constructs (Sup. Fig. 4). Expression of these in total lysate samples was also quantified (Sup. Fig. 5). P-Rex2 mutations included K254A (P-Rex1 K280A), R263A (P-Rex1 R289A), K337A (P-Rex1 K368A), and combinations thereof. Variants with mutations in the binding pocket were not deficient in membrane localization, even when all three critical PIP_3_-binding residues are altered (Fig. 4A). We next examined activity of these variants in HEK293T cells using a luciferase-gene reporter assay system that measures activation of GTPases such as Rac, Cdc42, and RhoA. Mutation of any of the PIP_3_-binding residues reduced P-Rex2 activity by 50% or greater, with R263A showing the largest effect (Fig. 4B and Sup. Fig. 6). Interestingly, although the trends in the effects of these mutations on activity are the same in P-Rex1 and P-Rex2, the extent of the effects (as compared to wild-type) is around two-fold less in P-Rex2 (Table 2). In fact, the triple point mutation in P-Rex1 results in an inactive protein (Cash et al., 2016), whereas the corresponding P-Rex2 variant still exhibits measurable activity. These results suggest that PIP_3_ binding is less important to P-Rex2 activity in cells relative to P-Rex1.

**Table 2:** Fold decrease in P-Rex variant activity. Data from the luciferase-reporter gene assays were used to calculate fold decreases in mean variant activities as compared to wild-type. For P-Rex1 variants, data published in Cash et al, *Structure* 2016 were used. Corresponding P-Rex2 and P-Rex1 mutations are shown on the same row.

P-Rex2		P-Rex1	
Mutation	Activity Fold Decrease	Mutation	Activity Fold Decrease
K254A	2	K280A	4
R263A	5	R289A	14
K337A	2.5	K368A	3
K254A/R263A	9	K280A/R289A	16
K254A/K337A	4	K280A/K368A	ND
R263A/K337A	8	R289A/K368A	14
K254A/R263A/K337A	15	K280A/R289A/K368A	28

ND, Not determined.

## 4 Discussion

Herein, we determined a high-resolution crystal structure of the P-Rex2 PH domain and showed that its PIP_3_-binding residues are important for P-Rex2 activity but not membrane localization in the context of the full-length protein in cells. Binding of PIP_3_-headgroup analogs to the PH domain are very similar in terms of thermal stabilization and affinity in P-Rex1 and P-Rex2. That said, PIP_3_ seems less important for P-Rex2 activation in cells because eliminating binding of the lipid had an attenuated effect on activation, as opposed to eliminating activity in P-Rex1.

We observed structural differences in the P-Rex1 and P-Rex2 PH domains that may underlie some of the differences in their regulation. P-Rex2 lacks a stretch of positively charged residues found in the β3/β4 loop of P-Rex1 that serves as a non-specific membrane localization element (Cash et al., 2016). This difference may lead to alternate modes of membrane recruitment between these two proteins. These could be through protein-protein or protein-lipid interactions mediated by the PH domain or by other domains of P-Rex. For example, DEP and PDZ domains are known to play roles in membrane association in other proteins. In DVL and EPAC, the DEP domains contain stretches of basic residues in β hairpins that are required for recruitment to the plasma membrane (Axelrod et al., 1998, Consonni et al., 2012), and in PDZ-RhoGEF, the PDZ domain is necessary and sufficient for its targeting to the apical membrane of polarized intestinal epithelial cells (Consonni et al., 2014). The difference in levels of P-Rex2 as compared to P-Rex1 at the cell membrane (Cash et al., 2016) is important when considering P-Rex activation and regulation, as the co-activator Gβγ also is localized there. Inherently higher levels of P-Rex2 at the cell membrane may be a consequence of a stronger association with Gβγ or another membrane-anchored molecule, leaving P-Rex2 comparatively less dependent on PIP_3_ for activation.

We also observed structural differences in the β5/β6 loop of the P-Rex2 PH domain. In P-Rex1, this loop has been proposed to be a protein-protein interaction site based on the fact that in most cases it forms the same anti-parallel dimeric lattice contact regardless of spacegroup or crystallization condition (Cash et al., 2016). Because this loop is structurally different in P-Rex2 but also forms a crystal contact site (Fig. 1 and Sup. Fig. 1), it remains a good candidate for protein-protein interactions, albeit ones that may be specific to this isoform. Interestingly, multiple mutations in the P-Rex2 PH domain, such as A315D, located in the β5/β6 loop, have been associated with lung adenocarcinoma (Srijakotre et al., 2017, Suzuki et al., 2013), suggesting the importance of this element in regulation, either auto-regulation or by other molecules. Structural information derived from larger fragments of P-Rex that contain the PH domain in the context of the other more C-terminal P-Rex domains, and their complexes with regulatory molecules, will thus be useful in furthering our understanding of the molecular mechanisms of P-Rex regulation.

## Declaration of interests

None.

## Funding information

This work was supported in part by 10.13039/100000002NIH grants HL071818, HL122416, and CA221289 to J.T. and an 10.13039/100000048American Cancer Society – Michigan Cancer Research Fund Postdoctoral Fellowship (PF-14-224-01-DMC) to J.C. This research used resources of the Advanced Photon Source, a U.S. Department of Energy (DOE) Office of Science User Facility operated for the DOE Office of Science by 10.13039/100006224Argonne National Laboratory under Contract No. DE-AC02-06CH11357. Use of the LS-CAT Sector 21 was supported by the 10.13039/100004948Michigan Economic Development Corporation and the Michigan Technology Tri-Corridor (Grant 085P1000817). This research used the DNA Sequencing Core of the Michigan Diabetes Research and Training Center supported by DK20572. The Center for Structural Biology, wherein ITC experiments were performed by Krishnapriya Chinnaswamy, is grateful for support from the Life Sciences Institute, the UM Comprehensive Cancer Center, the UM Medical School Endowment for Basic Sciences, and grants from the NIH.

## Author contribution statement

Conceptualization, J.C. and P.S.; Methodology, J.C. and P.S.; Investigation, J.C. and P.S.; Writing – Original Draft, J.C. and P.S.; Writing – Review & Editing, J.T. and J.C.; Funding Acquisition, J.T. and J.C.
