# Supplementary material for: Structural and biochemical characterization of the pleckstrin homology domain of the RhoGEF P-Rex2 and its regulation by PIP3
Source: J Struct Biol X. 2018 Dec 10;1:100001. doi: 10.1016/j.yjsbx.2018.100001 (PMC7337056; doi:10.1016/j.yjsbx.2018.100001)
Supplement: Supplementary data 1 [file mmc1.pdf]

Supplementary Data

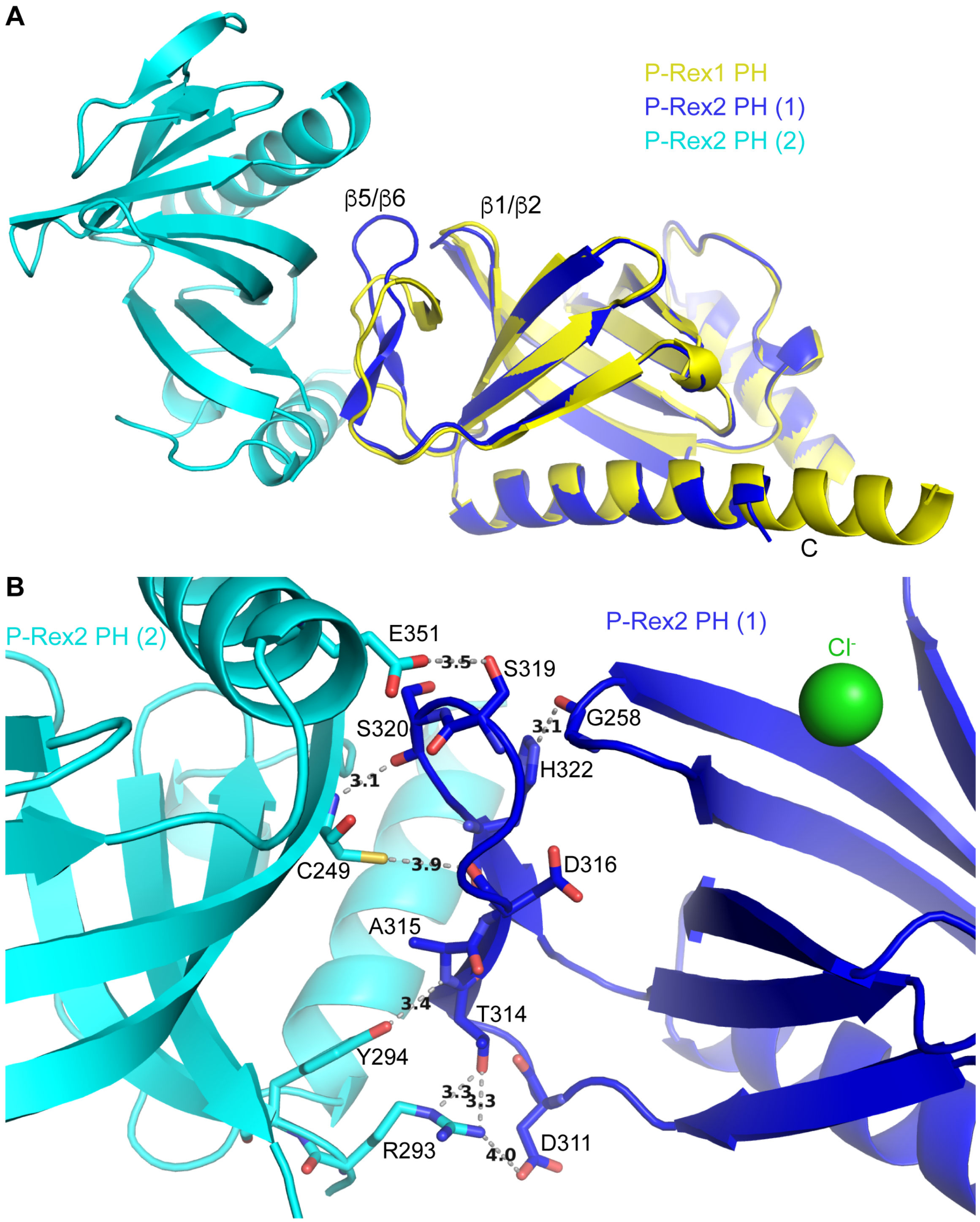

Supplementary Figure 1. Crystal contacts formed by the P-Rex2 β5/β6 loop. (**A**) One P-Rex1 PH domain from an Ins(1,3,4,5)P_4-bound structure (5D3X chain A, yellow) is aligned to the P-Rex2 PH structure (blue). A symmetry-related subunit from the P-Rex2 PH structure is shown (cyan) to illustrate the crystal packing at the β5/β6 loop, which exhibits conformational differences between P-Rex1 and P-Rex2. (**B**) Measurements between atoms within hydrogen bonding distance are shown (Å). A chloride ion occupying the PIP_3-binding site is depicted by a green sphere.

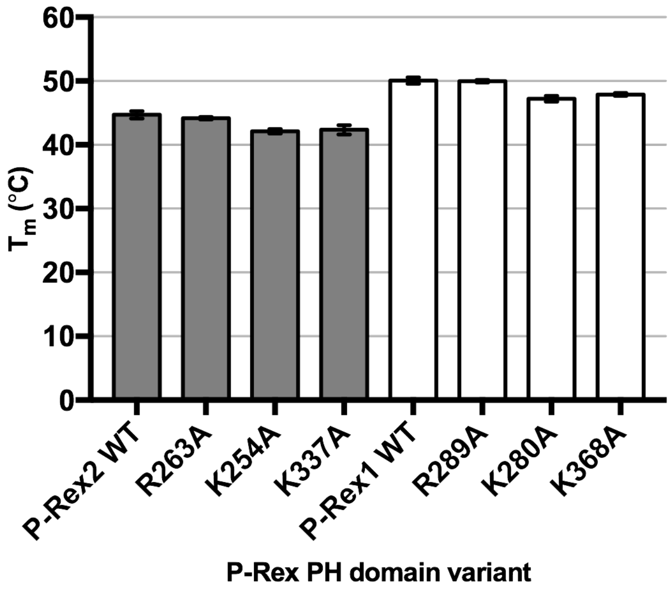

Supplementary Figure 2. Thermal stability of P-Rex PH domain variants. DSF was performed with wild-type and variant P-Rex PH domains, and melting temperatures (T_m) for each were determined. Experiments were performed three times in duplicate, and error bars represent 95% confidence intervals.

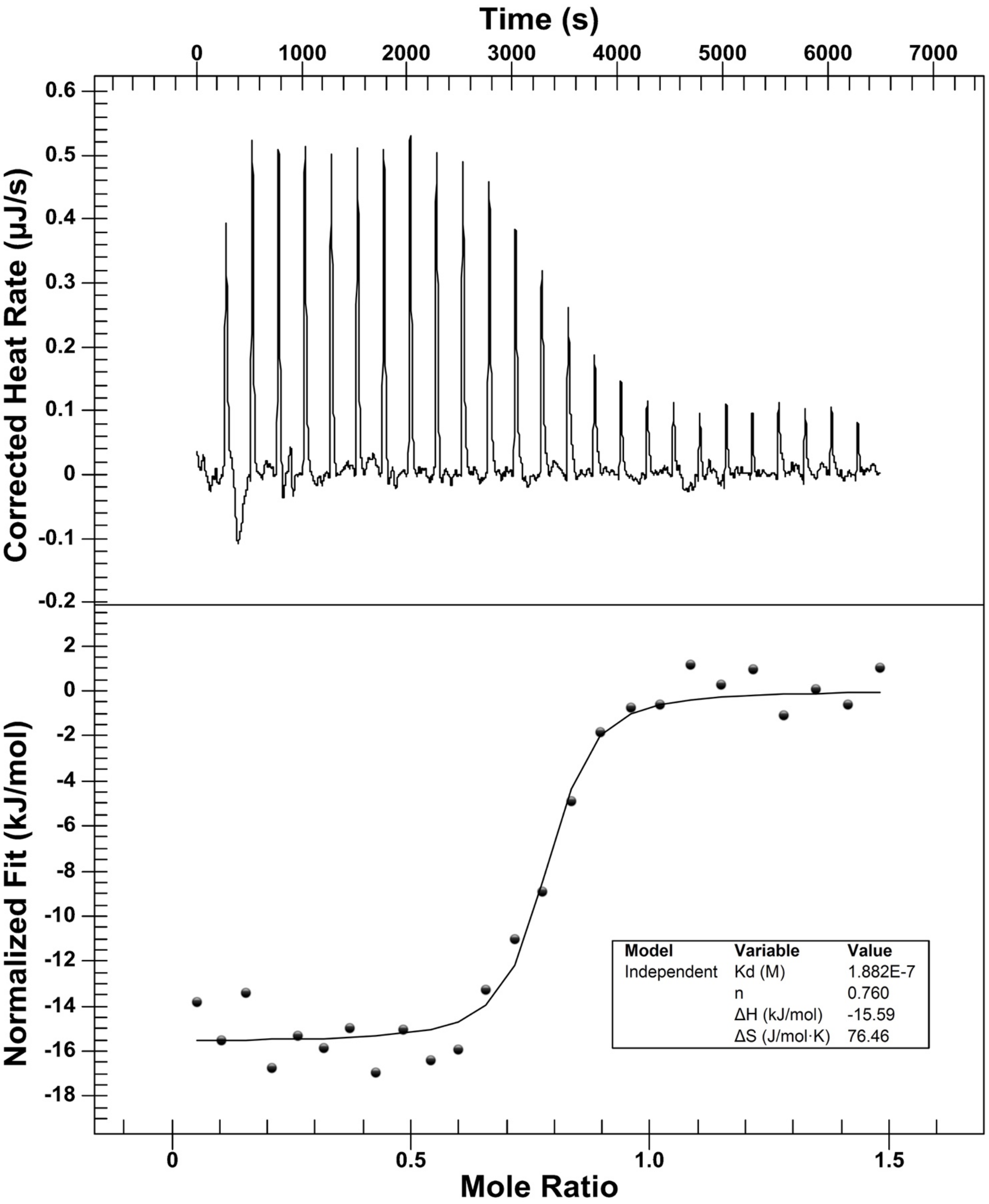

Supplementary Figure 3. Binding of Ins(1,3,4,5)P_4 to the P-Rex2 PH domain measured by isothermal titration calorimetry (ITC). ITC was performed as described in the Material and Methods section. Shown is one representative experiment out of three total.

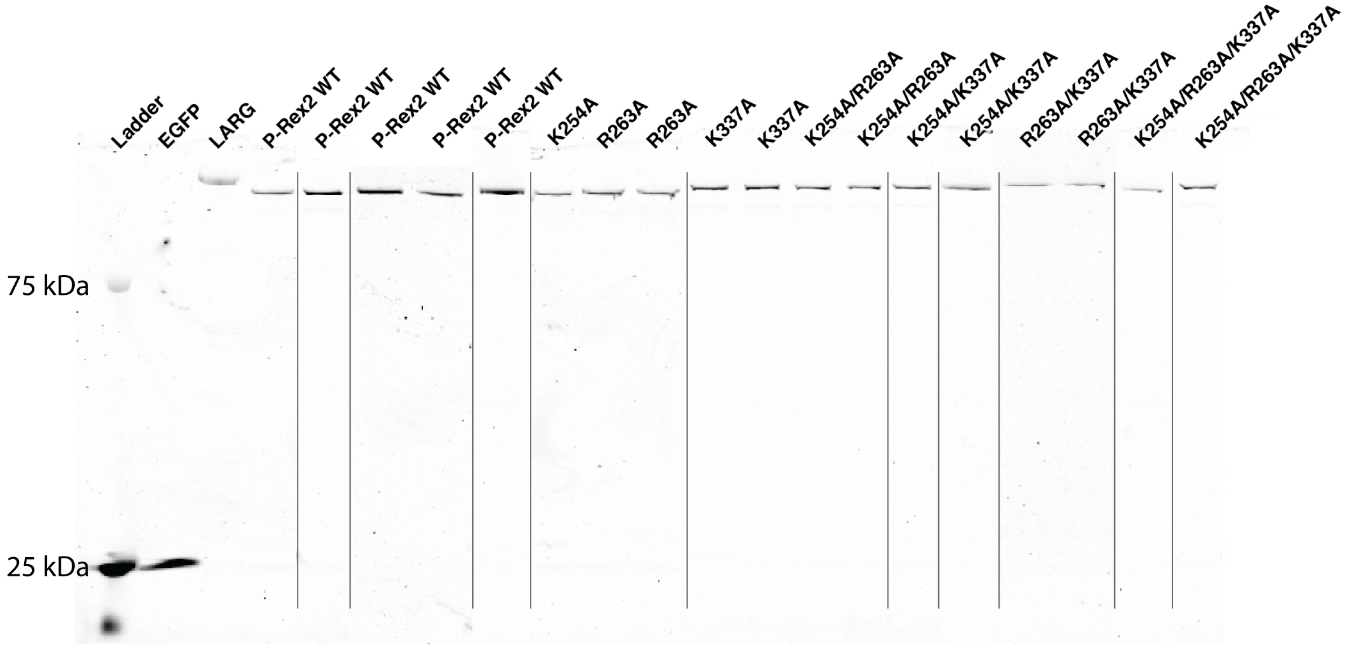

Supplementary Figure 4. EGFP-tagged P-Rex2 constructs used in activity and membrane localization assays run on SDS-PAGE. To verify that the majority of P-Rex2 proteins expressed in these experiments were full-length, a fraction of each total lysate sample from the membrane localization experiments was analyzed on SDS-PAGE, as outlined in the Material and Methods section. Shown are representative samples of each construct. A single band at the expected molecular weight was seen for each sample. This figure is a composite of multiple gel images placed side by side and aligned based on ladder bands. Vertical grey lines indicate were gel images were spliced together. The ladder contains EGFP (25 kDa) as well as a larger tagged protein that fluoresces at the same wavelength.

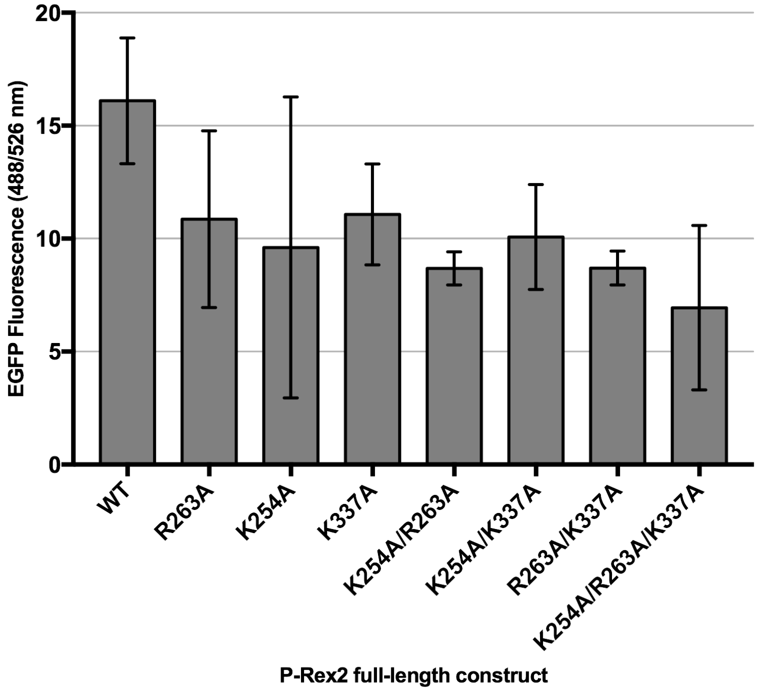

Supplementary Figure 5. Quantification of EGFP-tagged P-Rex2 construct expression. A fraction of each total lysate sample from the membrane localization experiments was analyzed for total EGFP fluorescence (RFU) to quantify relative levels of expression among P-Rex2 constructs. Error bars represent 95% confidence intervals.

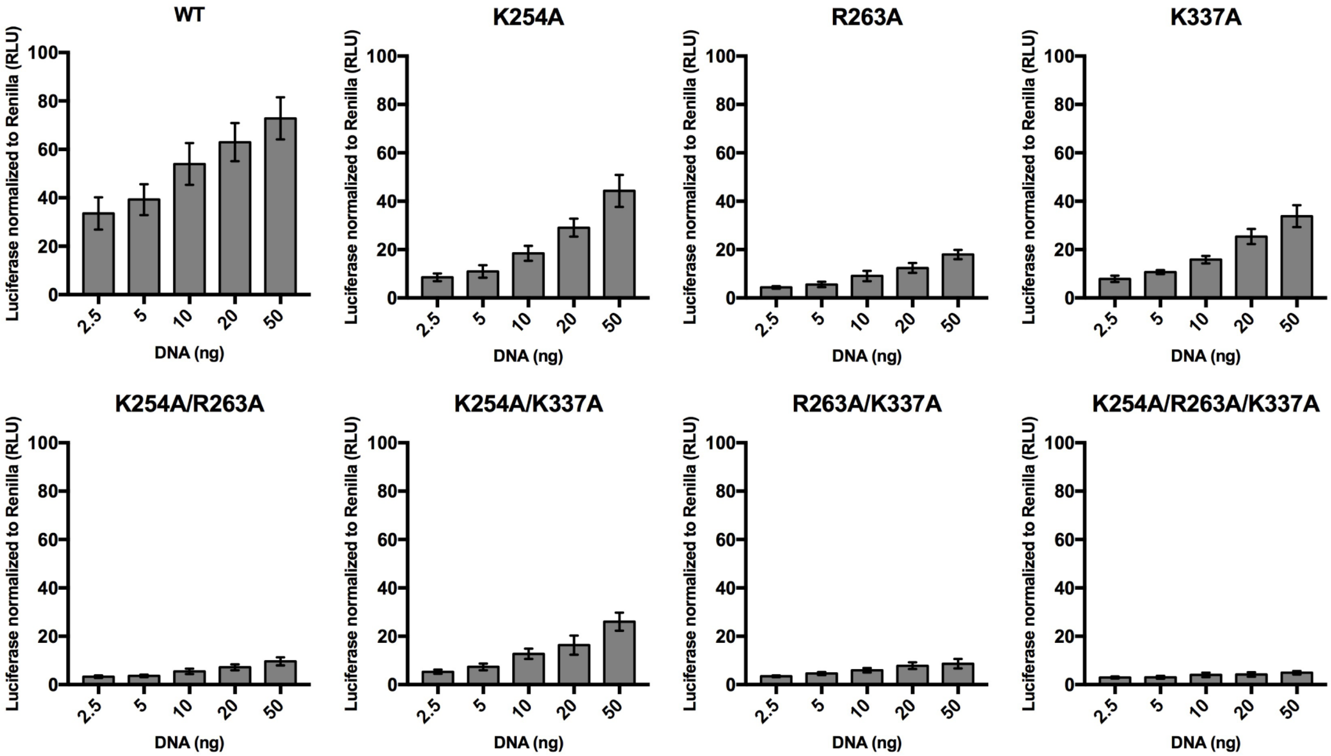

Supplementary Figure 6. DNA titrations for each construct used in the luciferase-reporter gene assays. To verify that each assay was working properly, a titration of each DNA construct was used for every experiment. The 20 ng data point is shown in Figure 4B for each construct to facilitate comparison. Experiments were performed at least four times in triplicate. Averages are shown with error bars representing 95% confidence intervals.
